# Detecting Forged Audio Files Using “Mixed Paste” Command: A Deep Learning Approach Based on Korean Phonemic Features

**DOI:** 10.3390/s24061872

**Published:** 2024-03-14

**Authors:** Yeongmin Son, Jae Wan Park

**Affiliations:** 1 Department of Digital Media, Soongsil University, Seoul 07027, Republic of Korea; 2 Global School of Media, Soongsil University, Seoul 07027, Republic of Korea

**Keywords:** Mixed Paste, Korean phonemic features, forged smartphone audio files, deep learning, audio forgery, transition band

## Abstract

The ubiquity of smartphones today enables the widespread utilization of voice recording for diverse purposes. Consequently, the submission of voice recordings as digital evidence in legal proceedings has notably increased, alongside a rise in allegations of recording file forgery. This trend highlights the growing significance of audio file authentication. This study aims to develop a deep learning methodology capable of identifying forged files, particularly those altered using “Mixed Paste” commands, a technique not previously addressed. The proposed deep learning framework is a composite model, integrating a convolutional neural network and a long short-term memory model. It is designed based on the extraction of features from spectrograms and sequences of Korean consonant types. The training of this model utilizes an authentic dataset of forged audio recordings created on an iPhone, modified via “Mixed Paste”, and encoded. This hybrid model demonstrates a high accuracy rate of 97.5%. To validate the model’s efficacy, tests were conducted using various manipulated audio files. The findings reveal that the model’s effectiveness is not contingent on the smartphone model or the audio editing software employed. We anticipate that this research will advance the field of audio forensics through a novel hybrid model approach.

## 1. Introduction

The widespread adoption of smartphones has led to the increased use of voice recording for personal and business applications [1]. Furthermore, audio editing tools such as Adobe Audition, Audacity, and iZotope have evolved beyond basic functionalities such as deletion, insertion, and connection. These tools now offer advanced spectrogram-based editing, enabling even laypeople to manipulate audio files without requiring professional editing skills [2,3]. This accessibility raises concerns as these forged audio files might be presented as evidence in legal proceedings. Thus, the detection of audio file forgeries has become a crucial area of research [3].

Audio file forgeries typically involve modifications using audio editing software, followed by adjustments to mimic the file structure and metadata of the original recording. These forgeries fall into two categories: container-based and content-based methods [2,4]. Container-based forgeries alter the file’s structure and metadata to match those of a device-recorded file. Tools such as MediaInfo [5], Hex editor [6], and MP4 inspector [7] can efficiently detect such manipulations. However, the proliferation of audio editing apps on smartphones allows for the easy replication of metadata and file structures mirroring the encoding of the recording device [8,9].

Content-based forgeries involve actual audio content manipulation using functions such as insertion, deletion, and connection. This category can be further divided into audio splicing forgery and audio copy-move forgery [2,4]. Audio splicing forgery involves the creation of a forged file by combining segments from two or more recordings. In contrast, audio copy-move forgery is executed by copying and pasting audio segments within the same file. Owing to the disparate sources of audio segments in splicing forgeries, various detection algorithms exploit differences such as microphone characteristics and environmental sounds to identify such forgeries [10,11,12,13]. Consequently, detecting audio splicing forgery tends to be simpler than identifying audio copy-move forgery [3,14]. Recent research has focused on employing deep learning for audio forgery detection [3,10,15,16,17]. However, these deep learning-based studies face a critical limitation: they depend on datasets that have not undergone the same encoding process as the recording device, potentially impacting their effectiveness. 

**Figure 1:**
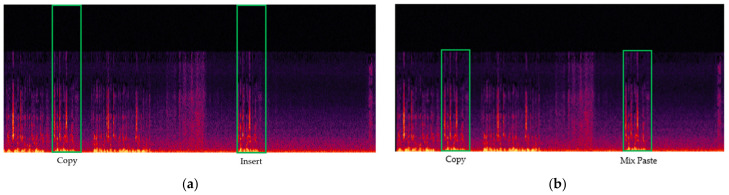
Audio copy-move forgery: (**a**) insertion; (**b**) mixed-paste.

Audio copy-move forgery can be classified into two types: insertion and mixed-paste. Figure 1 illustrates that audio copy-move forgery typically involves inserting a duplicated segment into a newly created timeframe. In contrast, mixed-paste forgery entails overlaying a segment onto an existing timeframe without generating a new one [18]. The tool enabling this process is referred to as the “Mix Paste” command. This command facilitates the overlapping of two speakers’ voices or the insertion of voices into an existing silent segment. The utilization of “Mix Paste” enhances the precision of forgery, thereby complicating its detection. To the best of our knowledge, no research has been conducted yet on “Mix Paste”-based audio copy-move forgery.

This study aims to develop a deep learning model capable of detecting audio forgeries executed using the “Mix Paste” command. The innovation lies in a novel method of feature extraction from spectrograms, focusing on the phonemes of Korean consonants. Utilizing this approach, we propose a hybrid deep-learning model that integrates a convolutional neural network (CNN) model and a long-short term memory (LSTM) model. The model’s training and testing are conducted using a dataset derived from Korean audio files recorded on an Apple iPhone 13 mini and an Apple iPhone 14 Pro Max, edited with the “Mix Paste” feature of Adobe Audition.

The hybrid model demonstrates a high accuracy rate of 97.5%. To comprehensively evaluate its performance, we conducted experiments with various forged files. These experiments indicate that the model’s effectiveness is not contingent on the type of audio editing software used or the iPhone model chosen for recording. Additionally, it proves capable of detecting audio splicing forgeries. However, the model has limitations, particularly with forged files that have a cutoff frequency differing from that of the dataset. It also cannot detect traditional insertion-based audio copy-move forgeries.

This research contributes significantly to the field of audio forensics by introducing a novel approach for feature extraction and constructing a deep learning-based hybrid model. It aims to enhance, broaden, and improve the detection capabilities for counterfeit audio files, addressing forgery techniques not previously considered in existing literature.

## 2. Proposed Method

### 2.1. Dataset

The dataset used in this study comprises Korean audio files manipulated using the “Mix Paste” command from Adobe Audition [19]. We have developed a genuine audio forgery dataset employing this technique and have made it publicly available for non-commercial purposes. Due to the differing frequency bandwidths and cutoff frequencies of iPhone and Samsung Galaxy smartphones, this dataset exclusively includes recordings made using iPhones. Considering the differences in frequency bandwidths and cutoff frequencies, iPhones provide a bandwidth of approximately 24 kHz and a cutoff frequency near 16 kHz. In contrast, Samsung Galaxy smartphones have a bandwidth of about 23 kHz with a higher cutoff frequency of around 20 kHz [19].

For dataset creation, a total of 933 audio files were recorded using the Apple iPhone 13 mini (451 files) and the Apple iPhone 14 Pro Max (482 files). These original audio files were subsequently edited using the “Mix Paste” command in Adobe Audition. In this process, genuine segments from one original file were copied and pasted into regions devoid of voice. Subsequently, the edited files were encoded using the iTunes encoder, which shares the same encoding system as the iPhone. Given that multiple forged files can be generated from a single recording, the dataset comprises 2604 forged files, including 1124 files recorded on the Apple iPhone 13 mini and 1480 files recorded on the Apple iPhone 14 Pro Max. The specifications of the dataset utilized in this study are detailed in Table 1.

**Figure 2:**
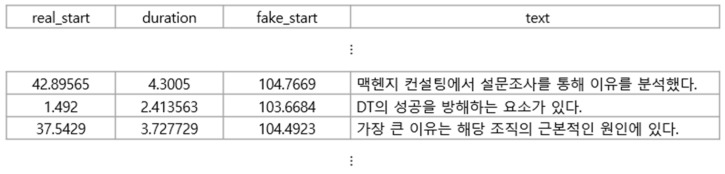
Time information and text in the CSV file: The Korean in the “text” column represents the contents of the audio file.

The recording script used to extract features from various Korean phonemes comprises plain, aspirated, sibilant, and tense consonants at 70.8%, 10.6%, 10.5%, and 8.1%, respectively. The contents of the audio file are converted into text by OpenAI’s open-source Whisper model [20] and stored. The time information and text of the segments are provided in a CSV file, as shown in Figure 2.

### 2.2. Feature Engineering

#### 2.2.1. Features of Korean Phoneme

The Korean language is characterized by a phonemic inventory consisting of 19 consonant and 21 vowel phonemes. Unlike English, Korean consonants are not categorized as voiced or voiceless, but by breath and tension into plain, tense, and aspirated consonants [21].

Plain consonants, the fundamental sounds in Korean consonants, include phonemes such as ㄱ[k/g] (giyeok), ㄴ[n] (nieun), ㄷ[t/d] (digeut), ㄹ[ɾ] (rieul), ㅁ[m] (mieum), ㅂ[p/b] (bieup), ㅇ[ŋ] (ieung), ㅈ[t͡ɕ/d͡ʑ] (jieut), andㅎ[h/ɦ] (hieut). These sounds are produced without significant breath force or throat strain. Tense consonants, comprisingㄲ[k͈] (ssanggiyeok), ㄸ[t͈] (ssangdigeut), ㅃ[p͈] (ssangbieup), ㅆ[s͈] (ssangsiot), and ㅉ[t͡ɕ͈] (ssangjieut), involve a closed glottis, increased pressure, and a sudden opening of the glottis, resulting in a pronounced explosive sound. These tense sounds are marked by greater pronunciation pressure and energy than plain sounds. Aspirated consonants, including ㅊ[t͡ɕʰ/d͡ʑʱ] (chieut), ㅋ[kʰ/ɡʱ] (kieuk), ㅌ[tʰ/dʱ] (tieut), and ㅍ[pʰ/bʱ] (pieup), are characterized by a forceful expulsion of air during articulation. The argument thatㅅ[sʰ] (siot) should be classified as an aspirated consonant due to its glottal spreading quality is supported by endoscopic and electromyography studies [22,23,24], suggesting it is closer to an aspirated sound than a plain one. In light of these phonetic characteristics, this study categorizes Korean consonants into four groups, as detailed in Table 2.

**Figure 3:**
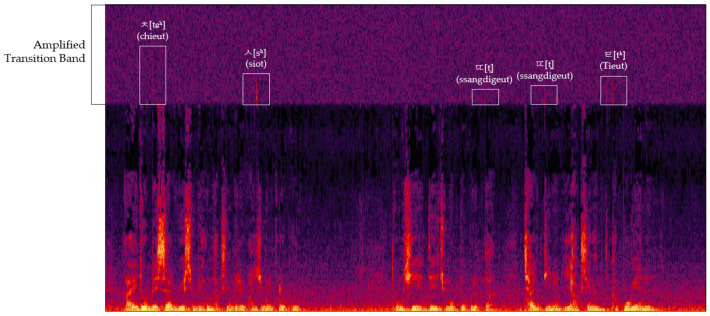
Phonemic traces in the amplified transition band.

Although individual variations in pronunciation exist, our study identified that aspirated, tense, and sibilant sounds leave distinct phonemic traces in the transition band of audio spectrograms. This band, characterized by a sharp roll-off between the pass-band and the stop-band, retains traces of these phonemes due to the inability of the low-pass filter to completely eliminate voice signals. For aspirated and sibilant sounds, the phonemic traces in the transition band are created by the air expelled during pronunciation. Similarly, tense sounds, known for their pronounced explosive onset, also leave identifiable traces in this band. By amplifying the transition band in a spectrogram, as demonstrated in Figure 3, these phonemic traces become observable. This phenomenon forms a crucial aspect of our study, offering insights into the unique acoustic characteristics of Korean consonants.

#### 2.2.2. Feature Extraction

**Figure 4:**
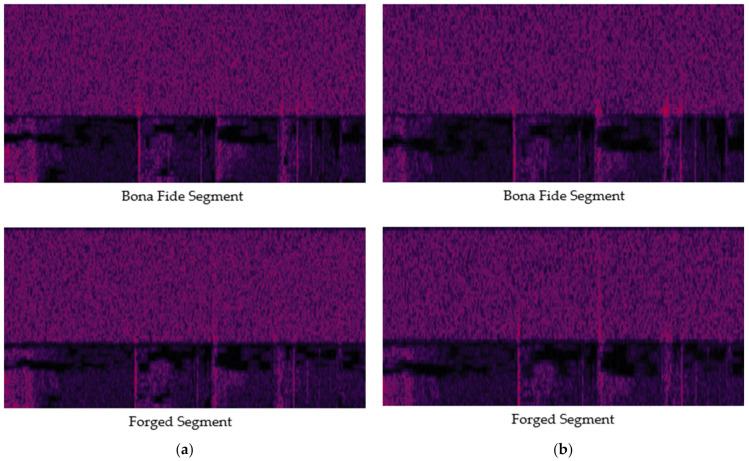
Comparison of phonemic traces generated in the transition band at sampling rates of (**a**) 48,000 Hz; (**b**) 44,100 Hz.

For an audio file to be admissible in court, its file structure and metadata must align with those of audio files from the same recording device. Therefore, the forged files in our dataset were encoded to match the original files’ sampling rate, set at 48,000 Hz. As depicted in Figure 4a, discernible differences between the authentic and forged segments were observed. However, when training the model with a dataset comprising audio files sampled at 48,000 Hz, the performance was suboptimal, achieving an accuracy rate of merely 58.47%. To improve this performance, we employed down-sampling to 44,100 Hz. Except for the down-sampling, we did not perform any data preprocessing steps such as noise reduction or silence removal. This adjustment effectively reduces the Nyquist frequency, leading to clearer and more pronounced phonemic traces, as illustrated in Figure 4b.

### 2.3. Model

#### 2.3.1. Model Building

The model developed in this study is a spectrogram-based hybrid model designed for the detection of audio file forgery, utilizing phoneme features. Hybrid models, which integrate different machine learning techniques, typically capitalize on the strengths and mitigate the weaknesses of each individual model. Such models are particularly prevalent in the field of speech analysis [25,26]. In our study, we combine the CNN model with the LSTM model, thereby effectively addressing both spectrogram images and pronunciation sequences.

The construction of our hybrid model involved several steps. Firstly, we employed a transfer learning-based CNN model using ResNet18 [27]. The choice of ResNet18, a pre-trained model, is due to its efficient learning structure for deep networks, which enables overcoming performance degradation issues in deep layered models [27]. Using the CNN block of our hybrid model, we extracted feature vectors of size 512 from the spectrograms as the output shape from the final average pooling layer in ResNet18 is 1 × 1 × 512.

Secondly, text was converted into a sequence of consonant types and processed through the LSTM. This conversion involved transforming graphemes to phonemes using g2pK, an open-source library [28], expressing the text based on phonological rules rather than grammatical rules. This was followed by the separation and symbolization of these phonemes into phonetic units using the open-source library KoG2P [29]. The consonants were subsequently categorized into plain, aspirated, tense, and sibilant sounds, forming a consonant sequence that the LSTM model processed. Due to its specialized neural network structure for handling sequential data, the LSTM could effectively learn deep and complex sequential data through a gate mechanism, making it suitable for use in hybrid models [30,31].

**Figure 5:**
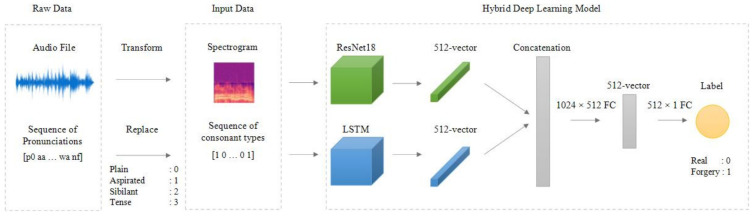
Model architecture.

Thirdly, we concatenated the 512-sized feature vector from ResNet18 with another 512-sized vector from the LSTM’s hidden state, resulting in a 1024-sized feature vector. This vector was passed through a 1024 × 512 fully connected (FC) layer and a 512 × 1 FC layer, and a sigmoid function subsequently classified it as original or forged audio. The architecture of this hybrid model is illustrated in Figure 5.

#### 2.3.2. Training

For the training and testing of the model, bona fide and forged audio segments were isolated. Among the 2604 forged audio files, those with editing errors were identified and removed through data inspection. Consequently, 2604 bona fide segments from original audio files and 2358 forged segments from forged audio files were selected for training and testing. To prevent data leakage, we assigned unique identifiers to our audio files. After dividing them into training and test sets, they were stored and managed on a separate solid-state drive (SSD).

**Figure 6:**
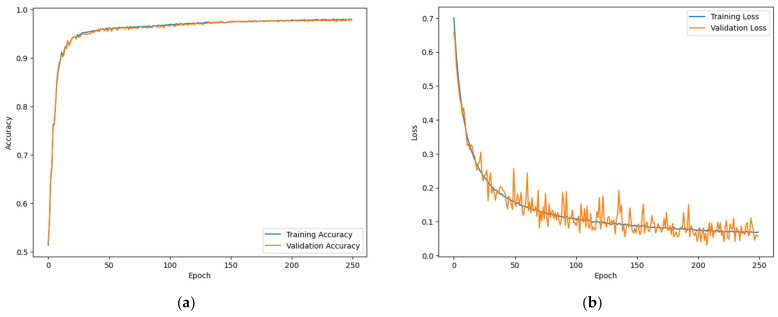
Learning curves: (**a**) training accuracy curve; (**b**) training loss curve.

These segments were allocated to separate training, validation, and test sets, divided in the ratio of 7:1:2. The training process was conducted on a system running Ubuntu 20.04, equipped with a GeForce RTX4090 graphics card. We used Python 3.10.9 and PyTorch 2.0.1 as the programming environment. The Adam optimizer was chosen for optimization, and Binary Cross Entropy was used as the loss function. The training parameters were set to 250 epochs, a learning rate of 0.005, and a batch size of 128. The learning curves, as depicted in Figure 6, indicate that the model underwent effective learning, adapting well to the training data.

#### 2.3.3. Results

When utilizing a Mel-spectrogram with a ResNet18-based transfer learning CNN model as a baseline, the model achieved a modest accuracy of 56%. This suggests that detecting audio files altered using the “Mixed Paste” technique with this baseline model is nearly impractical. However, the hybrid model developed in this study significantly outperformed this baseline, achieving a remarkable accuracy of 97.5%.

**Figure 7:**
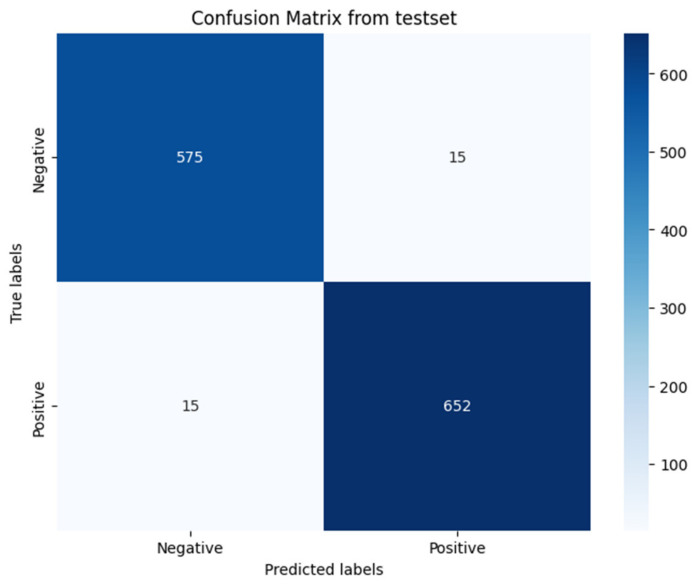
Confusion matrix.

The efficacy of the hybrid model is further illustrated in the confusion matrix presented in Figure 7. According to this matrix, out of 590 instances of original audio, the model correctly identified 575 as original, and out of 667 instances of forged audio, it correctly identified 652 as forged. Conversely, the model mistakenly classified 15 original audio files as forgeries and similarly misclassified 15 forged audio files as original.

**Figure 8:**
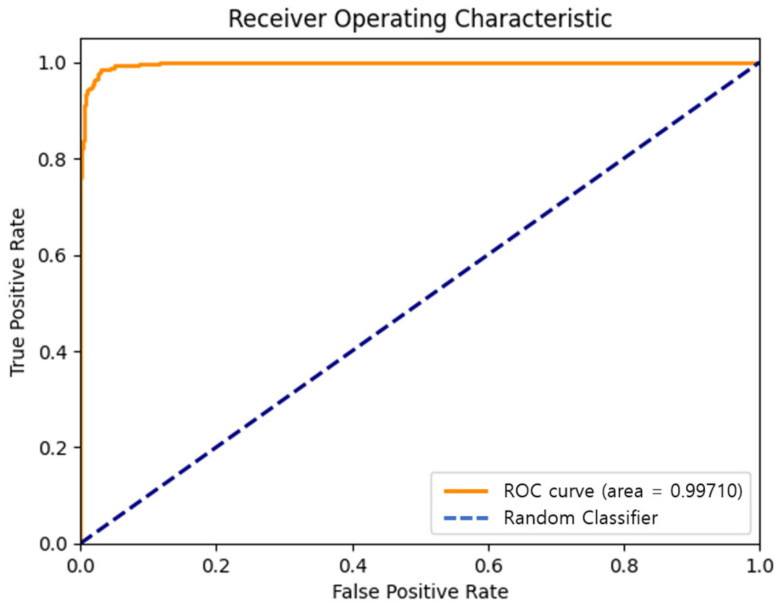
ROC curve.

From this confusion matrix, various performance metrics for binary classification were derived. These metrics are detailed in Table 3. Additionally, the receiver operating characteristic (ROC) curve, shown in Figure 8, provides a visual representation of the model’s performance across different thresholds, further validating the model’s high accuracy and robustness in distinguishing between original and forged audio files.

## 3. Audio Forgery Detection Experiments

To validate the performance of our hybrid model, we conducted a series of experiments addressing specific research questions:

1. Can the model detect forged files recorded on devices not used in building the dataset?
2. Is it capable of detecting forgeries created using editing software other than Adobe Audition?
3. Can it detect audio splicing forgery and insertion-based audio copy-move forgery?

For the first question, we tested our model’s ability to identify forgeries made from recordings on an iPhone 11, a device not included in the dataset. We created 368 forged audio files using “Mixed Paste” in Adobe Audition using iPhone 11 recordings and tested them with our model, which yielded an accuracy of 96%. Additionally, we evaluated the model’s performance on files recorded on a Samsung Galaxy Note 20, which has a different cutoff frequency (~20,000 Hz) compared with the iPhone (~16,000 Hz) used in training. This test showed a lower accuracy of 82%.

Answering the second question involved testing the model with forged files created using iZotope RX 10 [32], a different audio editing software. We constructed 198 forged files using the “Mixed Paste” command in iZotope RX 10 from original files recorded with the iPhone 14 Pro Max. Unlike Adobe Audition, iZotope RX 10 does not allow for specific part selection, leading to the inclusion of the transition band in the “Mixed Paste” command. This experiment resulted in a 95% accuracy rate.

To address the third question, we evaluated the model’s ability to detect splicing and insertion-based audio copy-move forgeries. We created 171 audio splicing forgery files by connecting two audio files recorded from the same device, achieving a high accuracy of 97%. However, in the detection of 200 audio copy-move forgery files created through insertion, the model’s accuracy dropped to 59%. The results of these experiments for the detection of different types of forged files are summarized in Table 4.

## 4. Discussion

The results from various forgery detection experiments indicate that our model is not confined to a specific audio editing software or iPhone model, and it is capable of detecting audio splicing forgeries. However, the model shows a reduced performance in detecting forgeries in audio files recorded using the Samsung Galaxy Note 20, with an accuracy of 82%; this device has a similar bandwidth frequency but a different cutoff frequency compared with the iPhones used in our dataset. This observation underscores the need to adopt additional research strategies to address and reduce these discrepancies.

Furthermore, the model is unable to detect traditional copy-move forgeries based on insertion, differentiating this method from “Mixed Paste” forgeries in terms of detectability. Additionally, a preliminary test with limited data revealed that the model can detect forgeries where another person’s voice is overlaid using the “Mix Paste” command, maintaining similar accuracy levels. However, when the same person’s voice overlapped, detection was not possible, likely due to different changes in the transition band than those previously observed; however, this situation is not practically possible.

This research is part of a larger, long-term project aimed at developing a system for detecting audio file forgeries that can be used in court. A critical consideration for court use is the model’s ability to avoid falsely identifying original files as forgeries. Although the specificity of our model currently stands at 97.4%, the specificity needs to be further improved without compromising sensitivity, ensuring the model’s reliability and applicability in legal contexts.

## 5. Conclusions

This study aimed to detect real forged audio files created using the “Mix Paste” command, a method not previously explored in the realm of audio copy-move forgeries. We introduced an innovative feature engineering technique focusing on Korean consonant phonemes, uncovering that aspirated, tense, and sibilant consonants exhibit subtle phoneme traces in the transition band of audio files. Utilizing these insights, we developed a hybrid model that combines a CNN with LSTM, based on ResNet18. This model demonstrated a high accuracy of 97.5%. The code developed in this study is available in the Supplementary Materials.

To evaluate the performance of the hybrid model, we conducted experiments with diverse forged files. The results indicated that our model is effective regardless of the specific audio editing software or iPhone models used. It was also shown to be successful in detecting audio splicing forgery. However, the model has limitations, notably its reduced performance, with forged files having different cutoff frequencies than those in the dataset, and its inability to detect traditional insertion-based audio copy-move forgeries. This suggests a need for separate research on traditional copy-move forgery techniques.

Future research directions, building on this study’s deep learning approach, include (1) enhancing performance through the development of additional datasets, (2) creating an insertion-based audio copy-move forgery dataset and developing a corresponding model, (3) constructing datasets for audio file forgery in other languages and their respective models, (4) optimizing model performance by integrating newer, potentially more efficient model structures, and (5) developing an improved algorithm capable of detecting forged audio files based on phonemic features.

This study is expected to make significant contributions to the field of audio forensics, particularly by offering a novel method for extracting phonemic feature points from speech and developing a deep learning model utilizing these features. Additionally, it advances the understanding of audio forgery detection methods by introducing a detection technique for “Mixed Paste” forgeries, a topic not previously addressed in the literature.

## 6. Patents

The fundamental concept of this paper is derived from the Korean patent “Apparatus for detecting forgery of Voice file and method thereof” (Registration Number: 10-2556425).

## Figures and Tables

**Table 1:** Specifications of the dataset used in the study.

Smartphone Model	Number of Original Files	Number of Forged Files	Sample Rate
Apple iPhone 13 mini Apple iPhone 14 Pro Max	451 482	1124 1480	48,000 Hz 48,000 Hz
Total	933	2604	-

**Table 2:** Classification of Korean consonant phonemes.

	Plain	Tense	Aspirated	Sibilant
Consonant Phonemes	ㄱ[k/g] (giyeok), ㄴ[n] (nieun), ㄷ[t/d] (digeut), ㄹ[ɾ] (rieul), ㅁ[m] (mieum), ㅂ[p/b] (bieup), ㅇ[ŋ] (ieung), ㅈ[t͡ɕ/d͡ʑ] (jieut),ㅎ[h/ɦ] (hieut)	ㄲ[k͈] (ssanggiyeok), ㄸ[t͈] (ssangdigeut), ㅃ[p͈] (ssangbieup), ㅆ[s͈] (ssangsiot), ㅉ[t͡ɕ͈] (ssangjieut)	ㅊ[t͡ɕʰ/d͡ʑʱ] (chieut), ㅋ[kʰ/ɡʱ] (kieuk), ㅌ[tʰ/dʱ] (tieut), ㅍ[pʰ/bʱ] (pieup),	ㅅ[sʰ] (siot) ^1^

^1^ In the consonant system of Korean standard language, it is included as a plain consonant.

**Table 3:** Performance metrics of binary classification.

	Accuracy (%)	Precision (%)	Recall (%)	Specificity (%)	F-1 Score (%)
Model	97.5	97.6	97.6	97.4	97.6

**Table 4:** Results of forgery audio detection experiments.

Dataset	Number of Forged Files	Accuracy (%)
Audio forged from iPhone 11 recording file using Mix Paste	368	96
Audio forged from Samsung Galaxy Note 20 recording file using Mix Paste	250	82
Audio files forged with iZotope RX	198	95
Audio splicing forgery	171	97
Insertion-based audio copy-move forgery	200	59

## Data Availability

Data are contained within the article.

## Supplementary Materials

Supporting information can be accessed at www.github.com/Dripmaster/audio-forgery-detection (accessed on 27 January 2024).
